# Supplementary material for: Evocalcet prevents ectopic calcification and parathyroid hyperplasia in rats with secondary hyperparathyroidism
Source: PLoS One. 2020 Apr 28;15(4):e0232428. doi: 10.1371/journal.pone.0232428 (PMC7188245; doi:10.1371/journal.pone.0232428)
Supplement: Attachment — Submitted filename: Response to Academic Editor.docx [file pone.0232428.s006.docx]

Dear Academic Editor

Tatsuo Shimosawa, M.D., Ph.D.

Academic Editor

PLOS ONE

Title: Evocalcet prevents ectopic calcification and parathyroid hyperplasia in rats with secondary hyperparathyroidism

Authors: Mariko Sakai, Shin Tokunaga, Mika Kawai, Miki Murai, Misaki Kobayashi, Tetsuya Kitayama, Satoshi Saeki, and myself.

We thank the referees for the carefully reviewing our manuscript and fruitful suggestions, especially for suggesting better terms and sentences concerning our manuscript. We found the comments of the reviewers most helpful and thus revised our manuscript according to the suggestions of the reviewers. We enclose revised versions of the manuscript, both with highlights of all changes and with unmarked changes. We also include a letter of our itemized responses to the Editor’s and Reviewer’s comments. We hope that this revised manuscript is now acceptable for publication in PLOS ONE.

About Funding and Competing Interests,

Mitsubishi Tanabe Pharma Corporation provided evocalcet. All studies were performed and the cost of them were supported by Kyowa Kirin Co., Ltd. Mariko Sakai, Shin Tokunaga, Mika Kawai, Miki Murai, Misaki Kobayashi, Tetsuya Kitayama, Satoshi Saeki, and Takehisa Kawata are employees of Kyowa Kirin Co., Ltd. Kyowa Kirin Co., Ltd provided support in the form of salaries for authors, MS, ST, MK, MM, MK, TK, SS and TK, but did not have any additional role in the study design, data collection and analysis, decision to publish, or preparation of the manuscript. The specific roles of these authors are articulated in the ‘author contributions’ section.

Evocalcet is the product in development, and Kyowa Kirin Co., Ltd and Mitsubishi Tanabe Pharma Corporation have the ownership of the patents of evocalcet.

These does not alter our adherence to PLOS ONE policies on sharing data and materials.

Thank you again for considering our manuscript. We are looking forward to hearing from you soon.

Respectfully yours,

Takehisa Kawata, PhD

Nephrology Research Laboratories, Nephrology R&D Unit, R&D Division

Kyowa Kirin Co., Ltd

1188, Shimotogari, Nagaizumi-cho, Sunto-gun, Shizuoka, 411-8731, Japan

Phone: +81-90-2047-4856

Fax: +81-55-986-7430

e-mail: [Takehisa.kawata.kk@kyowakirin.com](mailto:Takehisa.kawata.kk@kyowakirin.com)

Journal Requirements:

When submitting your revision, we need you to address these additional requirements.

1. Please ensure that your manuscript meets PLOS ONE's style requirements, including those for file naming. The PLOS ONE style templates can be found at

http://www.journals.plos.org/plosone/s/file?id=wjVg/PLOSOne_formatting_sample_main_body.pdf and <http://www.journals.plos.org/plosone/s/file?id=ba62/PLOSOne_formatting_sample_title_authors_affiliations.pdf>

We checked the format to make sure it was correct for PLOSONE style requirements. If there are any problems, please let us know.

2. Please include the total number of animals used in your study and the IACUC approval number related to your study. In the Methods section regarding the CKD rats with SHPT induced by 5/6 Nx please include the frequency of animal monitoring, including the specific criteria you used to monitor animal health.

Thanks for your helpful suggestions. We have noted animal numbers in each study in the method section (page 6, line 106, page 6, line 108, page 7, line 117, page 9, line 154).

We have also noted IACUC number as follows (page 6, line 98-102).

“protocol number APS 18J0188 for the single administration study, 17J0078 for the five-week administration study using CKD rats with SHPT induced by adenine, 14J0052 for the four-week administration study using CKD rats with SHPT induced by 5/6 Nx”

We revised the manuscript and described frequency of animal monitoring, including the specific criteria as follows (page 5, line 86-90).

“All experimental animals were monitored at least once per working day throughout the course of the study. Humane endpoints were defined as reduced physical activity level, weight loss, hunched posture, and other signs of distress. All rats reaching humane endpoints or in the single administration study were euthanized by carbon dioxide inhalation after the completion of studies.”

3. In regard to Evocalcet, please provide more information in the Methods regarding product lot number.

Thanks for your helpful comment. We have noted the lot number of evocalcet in the method section (page 5, line73-75).

4. We noticed you have some minor occurrence(s) of overlapping text with the following previous publication(s), which needs to be addressed:

https://doi.org/10.1371/journal.pone.0195316

In your revision ensure you cite all your sources (including your own works), and quote or rephrase any duplicated text outside the Methods section. Further consideration is dependent on these concerns being addressed.

I am very sorry that some of the expressions were duplicated from past our paper. We corrected the following points.

Page 3, line 50-54

“Cinacalcet is the first approved calcimimetic agent in 2004, allosterically modulates the calcium receptor (CaR) on parathyroid gland cells and suppresses PTH secretion [4, 5]. Cinacalcet has been widely used clinically for more than 10 years in the world for management of SHPT in dialysis patients [6-11] and succeeded to reduce the number of parathyroidectomies [12].”

Page 4, line 59-62

“Although cinacalcet has excellent PTH lowering effect, it has problems of causing upper gastro-intestinal (GI) side effects such as nausea and vomiting at a certain rate [17]. These problems sometimes become an obstacle to the long-term use of cinacalcet or treatment with increased doses of cinacalcet [18, 19].”

5. Thank you for stating the following in the Competing Interests/Financial Disclosure* (delete as necessary) section:

"The author(s) received no specific funding for this work."

We note that one or more of the authors are employed by a commercial company: Kyowa Kirin Co., Ltd..

1.	Please provide an amended Funding Statement declaring this commercial affiliation, as well as a statement regarding the Role of Funders in your study. If the funding organization did not play a role in the study design, data collection and analysis, decision to publish, or preparation of the manuscript and only provided financial support in the form of authors' salaries and/or research materials, please review your statements relating to the author contributions, and ensure you have specifically and accurately indicated the role(s) that these authors had in your study. You can update author roles in the Author Contributions section of the online submission form.

Please also include the following statement within your amended Funding Statement.

“The funder provided support in the form of salaries for authors [insert relevant initials], but did not have any additional role in the study design, data collection and analysis, decision to publish, or preparation of the manuscript. The specific roles of these authors are articulated in the ‘author contributions’ section.”

If your commercial affiliation did play a role in your study, please state and explain this role within your updated Funding Statement.

2. Please also provide an updated Competing Interests Statement declaring this commercial affiliation along with any other relevant declarations relating to employment, consultancy, patents, products in development, or marketed products, etc.

Within your Competing Interests Statement, please confirm that this commercial affiliation does not alter your adherence to all PLOS ONE policies on sharing data and materials by including the following statement: "This does not alter our adherence to PLOS ONE policies on sharing data and materials.” (as detailed online in our guide for authors http://journals.plos.org/plosone/s/competing-interests) . If this adherence statement is not accurate and there are restrictions on sharing of data and/or materials, please state these. Please note that we cannot proceed with consideration of your article until this information has been declared.

Please include both an updated Funding Statement and Competing Interests Statement in your cover letter. We will change the online submission form on your behalf.

Sorry for not enough information. Kyowa Kirin Co., Ltd. provided authors’ salaries and research fundings. We add following sentences at Disclosures section as follows (page 21, line 359-370).

“About Funding and Competing Interests, Mitsubishi Tanabe Pharma Corporation provided evocalcet. All studies were performed and the cost of them were supported by Kyowa Kirin Co., Ltd. Mariko Sakai, Shin Tokunaga, Mika Kawai, Miki Murai, Misaki Kobayashi, Tetsuya Kitayama, Satoshi Saeki, and Takehisa Kawata are employees of Kyowa Kirin Co., Ltd. Kyowa Kirin Co., Ltd provided support in the form of salaries for authors, MS, ST, MK, MM, MK, TK, SS and TK, but did not have any additional role in the study design, data collection and analysis, decision to publish, or preparation of the manuscript. The specific roles of these authors are articulated in the ‘author contributions’ section. Evocalcet is the product in development, and Kyowa Kirin Co., Ltd and Mitsubishi Tanabe Pharma Corporation have the ownership of the patents of evocalcet. These does not alter our adherence to PLOS ONE policies on sharing data and materials.”

6. We note that you have stated that you will provide repository information for your data at acceptance. Should your manuscript be accepted for publication, we will hold it until you provide the relevant accession numbers or DOIs necessary to access your data. If you wish to make changes to your Data Availability statement, please describe these changes in your cover letter and we will update your Data Availability statement to reflect the information you provide.

There are no clinical data, so we feel that this comment is not relevant to our manuscript.
